# Robertson on the Cause of Caries of the Teeth

**Published:** 1843-04

**Authors:** 


*Robertson on the Cause of Caries of the Teeth.*—Prior to the appearance of the first edition of Mr. Robertson's treatise, in 1835, the prevailing opinions concerning caries of the teeth were those of Fox and Bell; the former maintaining that the disease consists in an inflammation of the bony substance of the crown of a tooth, which secondarily affects the lining membrane of the organ, causing its separation, and a consequent decomposition of the solid parts; the latter insisting that caries commences in inflammation of the bone immediately under the enamel, from which, he says, the tooth, owing to its imperfect vitalization, cannot recover, and death and decay are the consequence.

Mr. Robertson, on the other hand, maintains that the remote cause of destruction of the teeth is the decomposition of food which lodges in the interstices between them.

The inflammatory theory, then, is to the effect that the teeth decompose by vital action, commencing in their interior, and proceeding to their surface. Our author's theory is exactly the opposite, namely, that decay begins by chemical action upon the surfaces of the teeth, and proceeds to their interior, and that inflammation is the consequence, and not the cause, of caries. His arguments are chiefly derived from the fact, that the teeth decay only in such situations as are favorable for the lodgment and decomposition of food, and never upon their smooth and even surfaces; from their decaying in pairs, and at particular periods of life; from the ready relief which filling and filing afford, if decay be not too far advanced; from the disease commencing externally and proceeding inwards, and never conversely; and from the circumstance, that artificial teeth are liable to the same species of destruction as natural ones.

These arguments we apprehend to be perfectly sound, and in accordance with the rules of common sense and experience; for we cannot imagine a disease, which owes its origin and progress to inflammation, to be susceptible of alteration or cure by means which, of all others, are most calculated to contribute to such action. But when the cause is purely chemical, and owing primarily to a defect of structure, it is not irrational to suppose that mechanical treatment will arrest or remedy it. It is on this ground that we think the practical application of Mr. Robertson's theory particularly valuable. Believing, as he does, that inflammation is not the cause of caries, but the consequence of it, and dependent upon the influence of atmospheric and other agencies upon the lining membrane of a tooth, he urges the necessity of removing any decay that may be apparent, *before the occurrence of pain;* for after its commencement there is little hope of permanent remedy. He insists upon the propriety of constantly using a tooth-brush, so that any particles of decomposing food may be removed; and of an occasional inspection of the teeth by a dentist, that any lurking decay may be arrested. He tells us that these suggestions have wrought a considerable change in his own practice—that people now apply to have caries arrested, rather than to have teeth removed in consequence of its ravages; and he maintains that, if this plan were universally acted upon, tooth-ache would be comparatively unknown, and the organs would be preserved in beauty and usefulness to the remotest period of life.—*Med. Ex.*, from *London Med. Gaz.*, Dec. 9, 1842.

